# Neoadjuvant chemoimmunotherapy was associated with better short‐term survival of patients with locally advanced esophageal squamous cell carcinoma compared to neoadjuvant chemoradiotherapy

**DOI:** 10.1002/cam4.70113

**Published:** 2024-08-13

**Authors:** Xiaofeng Duan, Fangdong Zhao, Xiaobin Shang, Jie Yue, Chuangui Chen, Zhao Ma, Zuoyu Chen, Chen Zhang, Qingsong Pang, Wencheng Zhang, Abbas E. Abbas, Hongjing Jiang

**Affiliations:** ^1^ Department of Minimally Invasive Esophageal Surgery Tianjin Medical University Cancer Institute and Hospital, National Clinical Research Center for Cancer, Tianjin's Clinical Research Center for Cancer, Key Laboratory of Cancer Prevention and Therapy Tianjin China; ^2^ Department of Radiation Oncology Tianjin Medical University Cancer Institute and Hospital Tianjin China; ^3^ Department of Thoracic Oncology, Lifespan Health System Warren Alpert Medical School of Brown University Providence Rhode Island USA

**Keywords:** chemoradiotherapy, esophageal squamous cell carcinoma, immunotherapy, neoadjuvant therapy

## Abstract

**Introduction:**

The chemotherapy and immunotherapy combination is currently the primary strategy to treat metastatic esophageal squamous cell carcinoma (ESCC). Neoadjuvant chemoimmunotherapy (NCIT) is being intensively investigated for treating locally advanced ESCC.

**Objective:**

We compared the efficacy and safety of NCIT and neoadjuvant chemoradiotherapy (NCRT) to treat locally advanced ESCC.

**Methods:**

We included 214 locally advanced ESCC patients who were administered neoadjuvant therapy from May 2014 to April 2022. The patients were grouped according to two neoadjuvant protocols (NCIT and NCRT) routinely used at our institution. Perioperative findings, pathological results, and survival data were compared between the two groups by conducting unmatched and 1:1 propensity score matching (PSM) analyses.

**Results:**

Following 1:1 PSM analysis of the confounders, 66 patients were allocated to each of the two groups. Time span between neoadjuvant therapy completion and esophagectomy was significantly longer after NCRT than that after NCIT (47.1 ± 13.2 days vs. 34.7 ± 8.8 days; *p* < 0.001). The NCIT group exhibited significantly greater number of harvested lymph nodes than the NCRT group (33.6 ± 12.7 vs. 21.7 ± 10.2; *p* < 0.001). The pathological complete response and major pathological response rates were similar between the two groups [NCIT group: 25.8% (17/66) and 62.1% (41/66), respectively; NCRT group: 27.3% (18/66) and 56.1% (37/66), respectively (*p* > 0.05)]. The overall incidence of pneumonia, anastomotic leakage, or postoperative complications did not differ significantly between the two groups. The 2‐year cumulative overall survival rates and the 2‐year disease‐free survival rates of the NCIT and NCRT groups were 80.2% and 62.2%, respectively (*p* = 0.029) and 70.0% and 50.8%, respectively (*p* = 0.023).

**Conclusion:**

In locally advanced ESCC patients, short‐term survival after NCIT is superior to that after NCRT, with similar perioperative and pathological outcomes.

## 1 INTRODUCTION

Esophageal cancer (EC), the seventh frequent malignant tumor globally, ranks sixth in cancer‐related death, and approximately 604,100 new EC cases and 544,076 deaths due to EC were reported in 2021.^1^ Esophageal squamous cell carcinoma (ESCC) is often detected in Eastern Asian countries, including China, while adenocarcinoma shows a higher prevalence in North America and Europe.

In the past two decades, the treatment approach for ESCC in China has changed from single modality to multimodality, which includes preoperative chemoradiotherapy or chemotherapy; this change has significantly improved patients' survival. In the CROSS study containing 23% patients with ESCC, the 5‐ and 10‐year overall survival (OS) rates after neoadjuvant chemoradiotherapy (NCRT) were 61.0% and 46%, respectively; these rates were remarkably higher than those of patients receiving surgery alone (30% and 23%, respectively).^2^ Furthermore, the median OS rates of the NCRT and surgery‐alone groups in the NEOCRTEC 5010 study were 100.1 and 66.5 months, respectively.^3^ Based on these studies, the current standard therapeutic approach is NCRT with subsequent surgery for locally advanced ESCC.

Despite this established approach, locoregional and distant recurrence continues to adversely affect the survival of ESCC patients for a longer term, even after multimodality therapy.^2^ The recurrence rate in the NEOCRTEC 5010 study following a median follow‐up period of 38.4 months was 33.7%, with distant metastasis and local recurrence rates of 19.6% and 9.8%, respectively, for patients receiving NCRT.^4^ These high failure rates prompted our group and other research groups to assess new treatment approaches for locally advanced ESCC.

According to recent studies, compared to chemotherapy alone, the combination treatment involving immunotherapy and chemotherapy can substantially enhance the progression‐free survival and OS of advanced ESCC patients.^5^, ^6^, ^7^, ^8^, ^9^ This combination is currently recommended as the primary treatment approach for metastatic and advanced ESCC. Applying chemotherapy and immunotherapy combination in the neoadjuvant chemoimmunotherapy (NCIT) is a novel and promising treatment modality.^10^ Based on several phase II studies, NCIT is both effective and safe for treating locally advanced ESCC.^11^, ^12^, ^13^ A meta‐analysis involving 815 patients in 27 clinical trials revealed the average pathological complete response (pCR) rate and the average rate of treatment‐related adverse events as 31.4% and 26.9%, respectively.^14^ Despite these encouraging outcomes, this new approach requires further validation.

The present retrospective study used propensity score matching (PSM) analysis to compare the treatment efficacy and safety of NCIT and NCRT for patients with locally advanced ESCC. Moreover, the perioperative outcomes and pathological responses were compared between these two groups, together with comparison of short‐term survival by utilizing the follow‐up data.

## 2 PATIENTS AND METHODS

### 2.1 Patients and surgical procedure

The Institutional Review Board of Tianjin Medical University Cancer Institute and Hospital granted approval to the study (no. bc2023135). All patients provided their informed consent to participate in the study. The study enrolled patients treated with neoadjuvant therapy and esophagectomy from May 2014 to April 2022 at our cancer center. Prior to the start date, neoadjuvant therapy was not routinely used in our institution. The surgical procedures and preoperative evaluation are reported earlier.^15^ For the present study, the inclusion criteria were (1) patients administered NCIT or NCRT, (2) patients who were diagnosed to have ESCC, and (3) patients who underwent three‐incision esophagectomy (McKeown procedure). The exclusion criteria were (1) patients with non‐SCC pathology, (2) patients receiving Ivor‐Lewis surgery due to insufficient lymph node dissection, and (3) patients who received preoperative chemotherapy alone.

For patients who underwent NCRT, radiotherapy (40–41.4 Gy, 20–23 fractions, dose: 1.8–2.0 Gy per fraction, frequency: 5 days/week) combined with chemotherapy was used. Chemotherapy regimens included the combination of a platinum agent and docetaxel, paclitaxel, or fluorouracil. The docetaxel plus cisplatin (TP) regimen included docetaxel (30 mg/m^2^ IV Q1W) or paclitaxel (50 mg/m^2^ IV Q1W) and platinum‐based drugs (nedaplatin or cisplatin: 25 mg/m^2^ IV Q1W; carboplatin: AUC 2 Q1W). The cisplatin plus fluorouracil regimen comprised platinum‐based drugs (75 mg/m^2^ IV Q3W) and 5‐fluorouracil (800 mg/m^2^ IV Q3W). The radiation dose was calculated based on intensity‐modulated radiotherapy validated by cone‐beam CT or image‐guided radiotherapy. Gross tumor volume (GTV) included the enlarged locoregional lymph nodes as well as the esophageal tumor. Clinical target volume (CTV) represented a 3 cm distal and proximal margin and a 6 mm radial margin around the GTV for including the subclinical involvement area. Planning target volume was estimated as a 5 mm margin around the CTV to compensate tumor movement and methodological variations. For the organs at risk, the following dose constraints were considered: lung V20 <30%, mean lung dose <18 Gy, heart V30 <40%, and spinal cord maximum dose <45 Gy.

Different immune checkpoint inhibitors inhibiting programmed cell death protein 1 (PD‐1) or PD‐1 ligand 1 (PD‐L1) were used as immunotherapeutic agents in combination with chemotherapeutic agents for the NCIT group. All patients who underwent NCIT were participating in clinical trials.^16^, ^17^, ^18^ The surgical procedures included lymph node dissection, transthoracic esophageal dissection, construction of the gastric tube, stomach mobilization, and left cervical esophagogastric anastomosis. Open, video‐assisted, and robotic‐assisted minimally invasive esophagectomy techniques were used. The technical specifics of the surgical procedure are described previously.^15^, ^19^

### 2.2 Data collection

The following data were collected: demographic data; cT, cN, and cTNM stage; neoadjuvant treatment data: NCIT protocols, radiotherapy dose, and chemotherapy regimens; interval between neoadjuvant therapy and surgery; surgical details (modality, blood loss, and operative time); postoperative hospital stay duration; postoperative mortality and morbidity rates; pathological data; and follow‐up data. The number of harvested lymph nodes was recorded from pathological reports. Major pathological response (MPR) was estimated to be ≤10% residual viable tumor in the primary lesion. Tumor regression grade (TRG) for esophageal tumor was estimated as follows: T1, no residual tumor; T2, residual tumor ≤10%; T3, residual tumor ≤50%; T4, residual tumor <100%; and T5, no tumor regression. The duration from incision to final closure was determined as the operative time, which also included time for body repositioning during the operation. OS was estimated as the period between neoadjuvant therapy initiation date to the date of death from any cause. Those patients who survived were censored at the last follow‐up date. The period between the surgery date and disease recurrence or death date was considered disease‐free survival (DFS). Disease recurrence was outlined as locoregional (regional lymph nodes or esophageal bed) or distant (distant organs [lungs, liver, brain, and bone] or distant lymph nodes). The Esophagectomy Complication Consensus Group criteria were followed to evaluate all major complications.^20^ The Tumor‐Node‐Metastasis Staging System 8th edition developed by the American Joint Committee on Cancer was used to stage all patients.^21^

### 2.3 Study endpoint

The primary study endpoints were short‐term survival, including 2‐year DFS and OS. The secondary endpoints were perioperative and pathological outcomes, including postoperative morbidity and mortality and pathological response.

### 2.4 Statistical analysis

Categorical variables were expressed as frequencies (%) and assessed by Fisher's exact test or the chi‐square test. Continuous variables were represented by mean ± standard deviation and assessed by Wilcoxon rank‐sum test or unpaired student's *t*‐test. A balanced cohort was formed using the available explanatory factors with the PSM approach. The original sample sizes for the patient pool were 136 and 78 for the NCRT and NCIT groups, respectively. The sample size available after matching was 66 for each group. Propensity scores were estimated by the logistic regression model. PSM (a 1:1 match for the NCIT and NCRT groups) was conducted blindly with a caliper distance of 0.1 and no replacement for adjusting identifiable factors that might influence the results, including age; sex; KPS score; smoking and drinking history; comorbidities; tumor location; and clinical T, N, and M stages. The Kaplan–Meier method was utilized for plotting OS and DFS curves, and the log‐rank test was used for comparing the curves. Statistical significance was considered at a two‐sided *p* < 0.05. All statistical analyses were conducted with SPSS version 25 for Windows (IBM SPSS Statistics, USA).

## 3 RESULTS

### 3.1 Patient demographics

**FIGURE 1:**
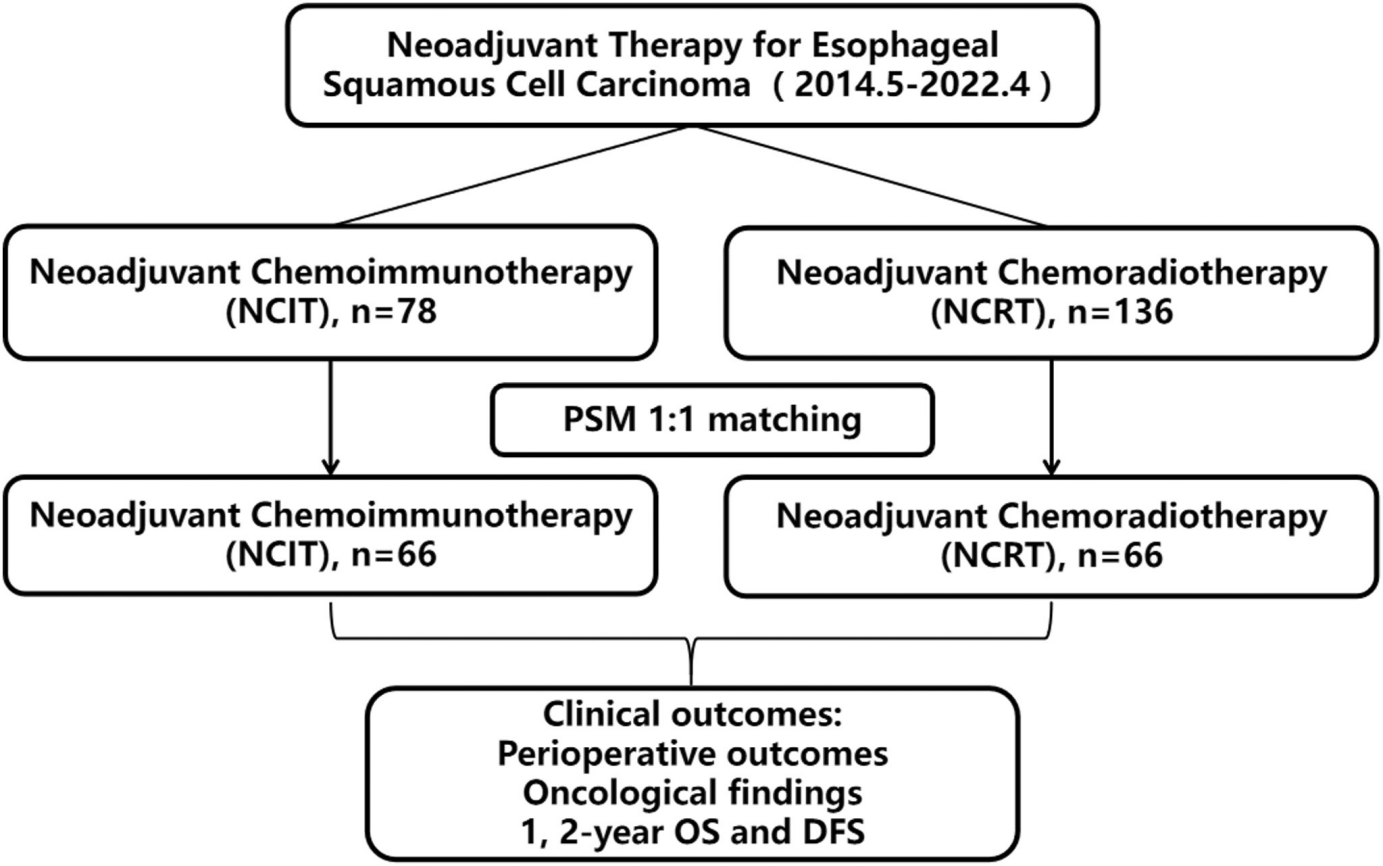
Study flow chart. A total of 214 patients were included in the present study, including 78 patients who received NCIT and 136 patients who received NCRT followed by surgery.

The study included 214 patients with locally advanced ESCC (Figure 1). After 1:1 PSM, 132 patients were obtained (66 per group). Table 1 shows the characteristics of unmatched and matched patients.

**TABLE 1:** Demographics variables.

	All patients *n* = 214 (%)	Before matching			After matching		
		NCIT, *n* = 78 (%)	NCRT, *n* = 136 (%)	*p* *	NCIT, *n* = 66 (%)	NCRT, *n* = 66 (%)	*p* *
Age, years, mean ± SD	59.7 ± 7.3	60.9 ± 7.3	59.1 ± 7.2	0.077	60.2 ± 7.3	58.2 ± 7.3	0.121
Sex ratio (M:F)	189:25	66:12	123:13	0.202	59:7	59:7	1.000
Smoking (*n*, %)	162 (75.7)	55 (70.5)	107 (78.7)	0.180	48 (72.7)	51 (77.3)	0.546
Drinking (*n*, %)	160 (74.8)	55 (70.5)	105 (77.2)	0.278	49 (74.2)	53 (80.3)	0.406
KPS score	88.7 ± 5.0	87.9 ± 5.2	89.1 ± 4.9	0.105	88.6 ± 4.9	89.4 ± 4.6	0.913
Comorbidity							
Hypertention	53 (24.9)	20 (26.0)	33 (24.3)	0.782	17 (25.8)	17 (25.8)	1.000
Diabetes	19 (8.9)	9 (11.5)	10 (7.4)	0.300	8 (12.1)	6 (9.1)	0.572
CHD	7 (3.3)	1 (1.3)	6 (4.4)	0.426	1 (1.5)	1 (1.5)	1.000
Tumor location							
20–25 cm	9 (4.2)	4 (5.1)	5 (3.7)	0.682	4 (6.1)	2 (3.0)	0.393
>25 and ≤30 cm	92 (43.0)	31 (39.7)	61 (44.9)		25 (37.9)	32 (48.5)	
>30 cm	113 (52.8)	43 (55.1)	70 (51.5)		37 (56.1)	32 (48.5)	
cT stage							
T1	3 (1.4)	3 (4.0)	0	<0.001	2 (3.0)	0	0.131
T2	3 (1.4)	0	3 (2.2)		0	3 (4.5)	
T3	142 (66.4)	68 (87.2)	74 (54.4)		57 (86.4)	59 (89.4)	
T4	66 (31.3)	7 (9.3)	59 (43.4)		7 (10.6)	4 (6.1)	
cN stage							
N0	30 (14.3)	10 (13.5)	20 (14.7)	0.339	9 (13.6)	9 (13.6)	0.293
N1	103 (48.1)	36 (46.2)	67 (49.3)		30 (45.5)	34 (51.5)	
N2	70 (33.3)	30 (40.5)	40 (29.4)		25 (37.9)	17 (25.8)	
N3	11 (5.2)	2 (2.7)	9 (6.6)		2 (3.0)	6 (9.1)	
cTNM stage							
II	17 (7.9)	7 (9.0)	10 (7.4)	<0.001	6 (9.1)	10 (15.2)	0.559
III	122 (57.0)	62 (79.5)	60 (44.1)		51 (77.3)	47 (71.2)	
IV	75 (35.0)	9 (11.5)	66 (48.5)		9 (13.6)	9 (13.6)	

*p<0.05 was considered as statistical significance.

### 3.2 Neoadjuvant therapy

Sixty‐two (79.5%) patients received pembrolizumab. Most patients (62/78, 79.5%) completed three cycles of NCIT. The majority of patients underwent concurrent NCRT (100/136, 73.5%). Paclitaxel+Platinum (TP)/Docetaxel+Platinum (DP) (110/136, 80.9%) was mainly administered as the chemotherapy regimen in the NCRT group (Table 2).

**TABLE 2:** Details of NCIT and NCRT.

NCIT	*n* = 78 (%)	NCRT	*n* = 136 (%)
Immunotherapy		Radiotherapy	
Pembrolizumab	62 (79.5)	Concurrent	100 (73.5)
Camrelizumab	7 (9.0)	Sequential	11 (8.1)
Sintilimab	6 (7.7)	Concurrent/sequential	22 (16.2)
Toripalimab	1 (1.3)	Radio doze	
Tislelizumab	1 (1.3)	<4000	4 (2.9)
Durvalumab	1 (1.3)	4000–4600	128 (94.1)
Chemotherapy		>4600	4 (2.9)
Paclitaxel+Cisplatin, TP	71 (91.0)	Chemotherapy	
Docetaxel+Cisplatin or Nedaplatin, DP	5 (6.5)	TP/DP	110 (80.9)
Docetaxel+Cisplatin+Fluorouracil, DCF	1 (1.3)	FP	10 (7.4)
Paclitaxel+Cisplatin+Fluorouracil, TCF	1 (1.3)	Others	16 (11.8)

### 3.3 Surgery

All patients underwent lymph node dissection and 3‐incision McKeown esophagectomy. After matching for baseline characteristics, the NCIT group had more patients who underwent robot‐assisted esophagectomy than the NCRT group (83.3% vs. 18.1%, *p* < 0.001). The time from completion of neoadjuvant therapy to surgery was significantly longer for the NCRT group (47.1 ± 13.2 days) than for the NCIT group (34.7 ± 8.8 days) (*p* < 0.001). Moreover, the NCIT group had significantly higher number of harvested lymph nodes than the NCRT group (33.6 ± 12.7 vs. 21.7 ± 10.2, *p* < 0.001).

### 3.4 Postoperative complications

Table 3 shows morbidity characteristics following esophagectomy in the matched and unmatched groups. The groups did not differ significantly in the incidence of total postoperative complications, either before matching (20.5% vs. 30.1%, *p* = 0.125) or after matching (21.2% vs. 31.8%, *p* = 0.168). Anastomotic leakage occurred in 3 (3.8%) and 17 (12.5%) patients in the NCIT and NCRT groups (*p* = 0.036), respectively, before matching. After matching, the NCIT and NCRT groups showed 3 and 7 leakages, respectively, with no significant difference (*p* = 0.188).

**TABLE 3:** Perioperative outcomes between NCIT and NCRT group.

	All patients *n* = 214 (%)	Before matching			After matching		
		NCIT, n = 78 (%)	NCRT, n = 136 (%)	*p* *	NCIT, *n* = 66 (%)	NCRT, *n* = 66 (%)	*p* *
Surgical procedure							
Open	44 (20.6)	3 (3.8)	41 (30.1)	<0.001	3 (4.5)	20 (30.3)	<0.001
MIE	75 (35.0)	10 (12.8)	65 (47.8)		8 (12.1)	34 (51.5)	
Robot‐assisted MIE	95 (44.4)	65 (83.3)	30 (22.1)		55 (83.3)	12 (18.1)	
Operation time, min, mean ± SD	313.0 ± 60.5	315.8 ± 60.0	311.4 ± 61.0	0.615	319.3 ± 63.2	308.9 ± 58.1	0.341
Surgical blood loss, ml, mean ± SD	180.3 ± 106.0	165.7 ± 143.2	189.0 ± 75.2	0.133	172.9 ± 149.7	185.9 ± 86.1	0.555
Surgical interval	43.0 ± 12.6	35.2 ± 9.0	47.6 ± 12.1	<0.001	34.7 ± 8.8	47.1 ± 13.2	<0.001
Lymph node harvest, mean ± SD	25.8 ± 12.6	34.4 ± 12.5	23.8 ± 21.1	<0.001	33.6 ± 12.7	21.7 ± 10.2	<0.001
Hospital stay, days, mean ± SD	20.5 ± 18.5	14.8 ± 10.7	23.8 ± 21.1	0.001	14.8 ± 11.0	24.7 ± 23.6	0.002
ICU stay, n (%)	13 (6.1)	3 (3.8)	10 (7.4)	0.383	3 (4.5)	3 (4.6)	1.000
Overall complications	57 (26.6)	16 (20.5)	41 (30.1)	0.125	14 (21.2)	21 (31.8)	0.168
RLN paralysis	5 (2.3)	4 (5.1)	1 (0.7)	0.060	4 (6.1)	1 (1.5)	0.365
Pulmonary complication	18 (8.4)	5 (6.4)	13 (9.6)	0.424	4 (6.1)	4 (6.1)	1.000
Anastomotic leakages	20 (9.3)	3 (3.8)	17 (12.5)	0.036	3 (4.5)	7 (10.6)	0.188
Chylothorax	8 (3.7)	2 (2.6)	6 (4.4)	0.713	1 (1.5)	4 (6.1)	0.365
irAEs	2 (0.9)	2 (2.6)	0	–	2 (2.9)	0	–
rrAEs	1 (0.5)	0	1 (0.7)	–	0	1 (1.2)	–
30‐day mortality	2 (0.9)	1 (1.3)	1 (0.7)	1.000	0	0	–
90‐day mortality	7 (3.3)	3 (3.9)	4 (2.8)	0.707	1 (1.5)	1 (1.5)	1.000

*p<0.05 was considered as statistical significance.

### 3.5 Pathological response

Among the 78 patients receiving NCIT, 47 (60.3%) and 20 (25.6%) patients exhibited MPR for primary tumor and pCR (ypT0N0), respectively. Among the 136 patients who underwent NCRT, 76 (55.9%) and 38 patients (27.9%) showed MPR and pCR, respectively. The NCIT and NCRT groups did not differ in terms of MPR, pCR, and TRG before or after PSM (*p* > 0.05; Table 4).

**TABLE 4:** Pathological Response between NCIT and NCRT group.

	All patients *n* = 214 (%)	Before matching			After matching		
		NCIT, *n* = 78 (%)	NCRT, *n* = 136 (%)	*p* *	NCIT, *n* = 66 (%)	NCRT, *n* = 66 (%)	*p* *
pT stage							
T0	81 (37.9)	31 (39.7)	50 (36.8)	0.093	26 (39.4)	25 (37.9)	0.202
T1	28 (13.1)	16 (20.5)	12 (8.8)		13 (19.7)	5 (7.6)	
T2	32 (15.0)	10 (12.8)	22 (16.2)		10 (15.2)	11 (16.7)	
T3	55 (25.7)	17 (21.8)	38 (27.9)		15 (22.7)	19 (28.8)	
T4	18 (8.4)	4 (5.1)	14 (10.3)		2 (3.0)	6 (9.1)	
pN stage							
N0	126 (58.9)	38 (48.7)	88 (64.7)	<0.001	33 (50.0)	41 (62.1)	<0.001
N1	59 (27.6)	22 (28.2)	37 (27.2)		17 (25.8)	21 (31.8)	
N2	15 (7.0)	15 (19.2)	0		14 (21.2)	0	
N3	14 (6.5)	3 (3.8)	11 (8.1)		2 (3.0)	4 (6.1)	
ypTNM stage							
I	95 (44.4)	33 (42.3)	62 (45.6)	0.008	29 (43.9)	29 (43.9)	0.333
II	23 (10.7)	4 (5.1)	19 (14.0)		4 (6.1)	9 (13.6)	
III	75 (35.0)	37 (47.4)	38 (27.9)		30 (45.5)	23 (34.8)	
IV	21 (9.8)	4 (5.1)	17 (12.5)		3 (4.5)	5 (7.6)	
pCR (ypT0N0)	58 (27.1)	20 (25.6)	38 (27.9)	0.716	17 (25.8)	18 (27.3)	0.844
MPR	123 (57.5)	47 (60.3)	76 (55.9)	0.533	41 (62.1)	37 (56.1)	0.479
TRG							
1	85 (39.7)	35 (44.9)	50 (36.8)	0.668	31 (47.0)	25 (37.9)	0.763
2	38 (17.8)	12 (15.4)	26 (19.1)		10 (15.2)	12 (18.2)	
3	21 (9.8)	8 (10.3)	13 (9.6)		7 (10.6)	6 (9.1)	
4	20 (9.3)	5 (6.4)	15 (11.0)		4 (6.1)	7 (10.6)	
5	50 (23.4)	18 (23.1)	32 (23.5)		14 (21.2)	16 (24.2)	

*p<0.05 was considered as statistical significance.

### 3.6 Survival

**FIGURE 2:**
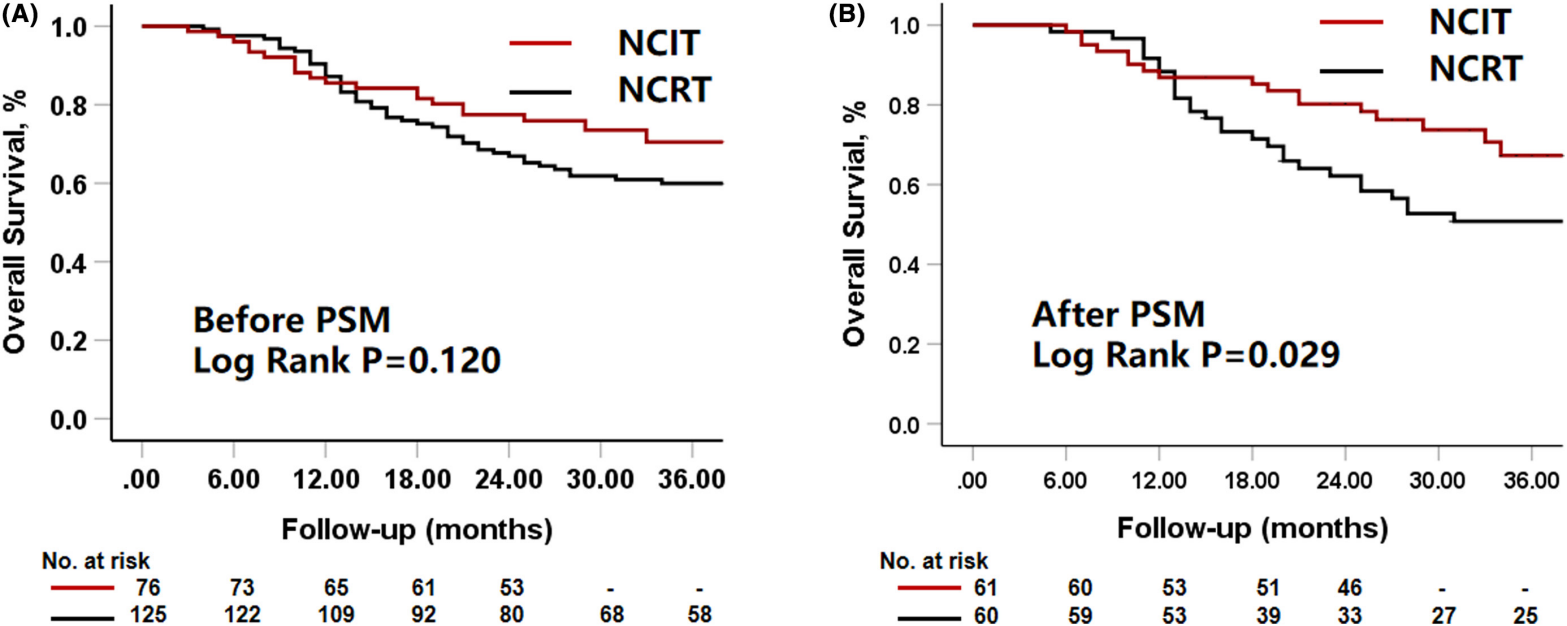
Overall survival (OS). (A) Before propensity score matching (PSM), the cumulative 1 and 2‐year OS rates were 85.5% and 77.5% in the NCIT group and 87.2% and 66.9% in the NCRT group, respectively (log‐rank test, *p* = 0.140). (B) After matching, the 1 and 2‐year cumulative OS rates were 86.9% and 80.2% in the NCIT group and 88.3% and 62.2% in the NCRT group, respectively (log‐rank test, *p* = 0.029).

Eleven patients were lost to follow‐up (9 and 2 patients in the NCRT and NCIT groups, respectively). The median follow‐up duration was 33 months (range: 4–96 months) and 27 months (range: 3–43 months) in the NCRT and NCIT groups, respectively. Before PSM, the cumulative 1‐ and 2‐year OS rates were 85.5% and 77.5% for the NCIT group and 87.2% and 66.9% for the NCRT group, respectively (log‐rank test, *p* = 0.140) (Figure 2A). As shown in Figure 2B, after matching, the 1‐ and 2‐year cumulative OS rates were 86.9% and 80.2% for the NCIT group and 88.3% and 62.2% for the NCRT group, respectively (log‐rank test, *p* = 0.029). Seven patients died due to other causes during the follow‐up period (1 from depression, 3 from acute myocardial infarction and heart failure, 1 from intestinal obstruction due to colon cancer at 2 years after esophageal surgery, 1 from trauma, and 1 from cholangitis), the 1‐ and 2‐year disease‐specific survival was 86.9% and 81.8% in the NCIT group and 88.3% and 63.5% in the NCRT group, respectively (log‐rank test, *p* = 0.043).

**FIGURE 3:**
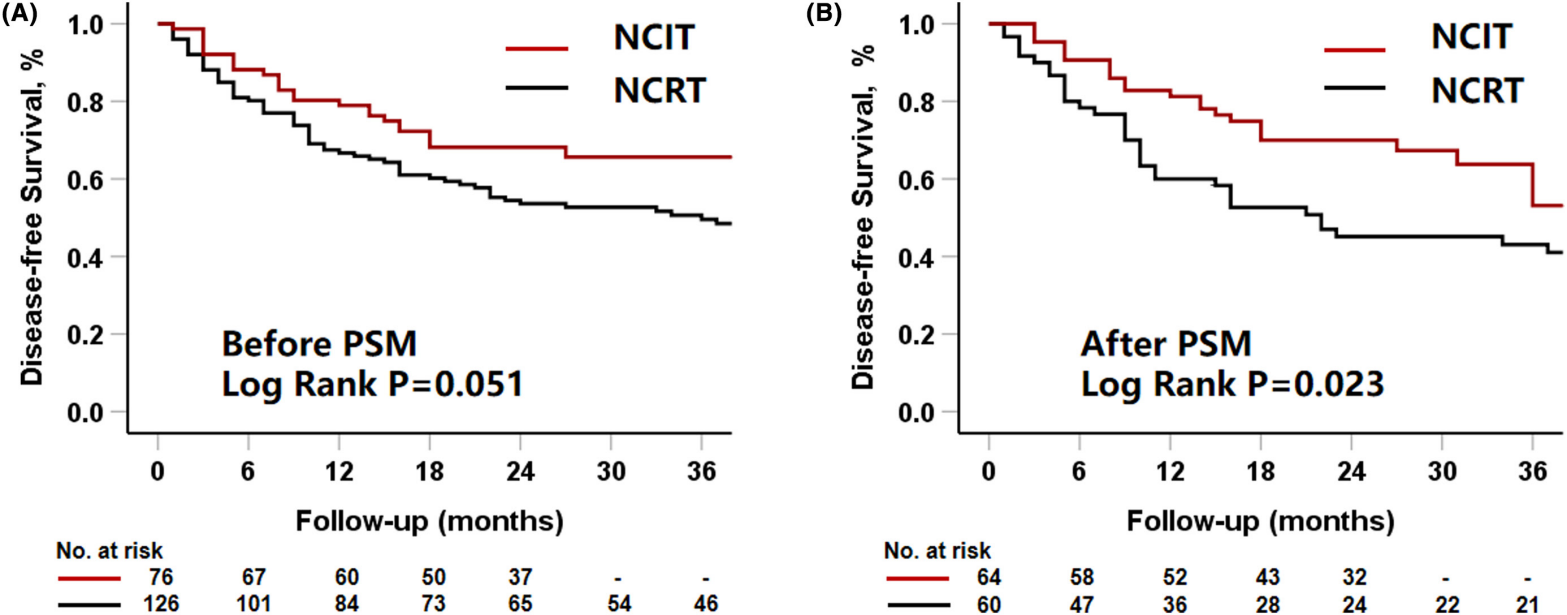
Disease‐free survival (DFS). (A) Before propensity score matching (PSM), the cumulative 1 and 2‐year DFS rates were 78.9% and 68.2% in the NCIT group and 66.7% and 53.6% in the NCRT group, respectively (log‐rank test, *p* = 0.051). (B) After matching, the 1 and 2‐year DFS rates were 81.3% and 70.0% in the NCIT group and 63.3% and 50.8% in the NCRT group, respectively (log‐rank test, *p* = 0.023).

Before matching, the 1‐ and 2‐year DFS rates were 78.9% and 68.2% in the NCIT group and 66.7% and 53.6% in the NCRT group, respectively (log‐rank test, *p* = 0.051) (Figure 3A). After matching, these rates were 81.3% and 70.0% in the NCIT group and 63.3% and 50.8% in the NCRT group, respectively (log‐rank test, *p* = 0.023) (Figure 3B).

Within the first year after surgery, death due to tumor occurred in 23 (18.1%) and 5 (6.6%) patients in the NCRT and NCIT groups, respectively, before PSM (*p* = 0.045). After PSM, the death rate from tumors was 21.7% (*n* = 13) and 4.6% (*n* = 3) in the NCRT and NCIT group, respectively (*p* = 0.013). The local and distant recurrence rates were 11.7% (*n* = 7) and 13.3% (*n* = 8) in the NCRT group and 6.2% (*n* = 4) and 1.5% (*n* = 1) in the NCIT group, respectively (*p* = 0.032). The recurrence details are shown in Table 5.

**TABLE 5:** Death and disease recurrence details within 1 year after surgery.

	Before matching			After matching		
	NCIT	NCRT	*p* *	NCIT	NCRT	*p* *
Cause of death	*n* = 76 (%)	*n* = 127 (%)	0.045	*n* = 65 (%)	*n* = 60 (%)	0.013
Tumor	5 (6.6)	23 (18.1)		3 (4.6)	13 (21.7)	
Treatment complications	5 (6.6)	4 (3.1)		4 (6.2)	1 (1.7)	
Others	2 (2.6)	1 (0.8)		2 (3.1)	1 (1.7)	
Disease recurrences	*n* = 76 (%)	*n* = 126 (%)	0.095	*n* = 65 (%)	*n* = 60 (%)	0.032
Local	5 (6.6)	15 (11.9)		4 (6.2)	7 (11.7)	
Distant	1 (1.3)	11 (8.7)		1 (1.5)	8 (13.3)	
Local + distant	1 (1.3)	4 (3.2)		1 (1.5)	3 (5.0)	
Death	9 (11.9)	12 (9.5)		7 (10.8)	6 (10.0)	

*p<0.05 was considered as statistical significance.

## 4 DISCUSSION

The present retrospective case–control study clarified the efficacy of anti‐PD‐1/anti‐PD‐L1 antibody combination with chemotherapy to treat locally advanced ESCC; the pCR rate was 25.8%, identical to the rate achieved with chemoradiotherapy. More significantly, the results for short‐term survival with NCIT were superior to those with NCRT with regard to 2‐year OS (80.2% vs. 62.2%) and DFS (70.0% vs. 50.8%).

Several studies are actively investigating neoadjuvant therapy for ESCC. Although the NCRT group had a higher pCR rate in the JCOG1109 study, a higher survival benefit was not achieved. Following the beginning of the immunotherapy era, neoadjuvant immunotherapy has become the focus of research to further improve the clinical outcomes of EC patients. A recent meta‐analysis summarized the safety and efficacy of NCIT for ESCC.^22^ Interestingly, Yin et al.^23^ recently published their observations of a phase 1b clinical trial involving neoadjuvant monotherapy with adebrelimab (a PD‐L1 blocker) in 30 patients with resectable ESCC. Of these patients, 24% had MPR; the pCR rate and 2‐year OS were 8% and 92%, respectively. Despite these impressive results, the standard therapy for locally advanced ESCC is NCRT with subsequent surgery.

In the present study, in the NCIT group, 60.3% patients (47 of 78 patients) had MPR of the primary tumor (TRG1‐2), and 25.6% showed complete resolution of both tumor and lymph node disease (pCR); this was comparable to that of 55.9% (MPR) and 27.9% (pCR) in the NCRT group. Similar pCR and primary tumor regression rates were observed after matching. The two groups differed in lymph node and tumor regression. The downstaging of N status was substantially more after NCRT. This may be because T cells and cytotoxic T cells exhibit differences in their in situ immune patterns between metastatic lymph nodes and primary tumors, as reported for other tumors such as lung cancer. This heterogeneity might lead to varied immune responses to the type of neoadjuvant immunotherapy.^24^ Further studies are warranted to clarify this difference in the immune pattern responses to neoadjuvant therapy.

The pCR rate of NCRT in our study (27.9%) was lower than that in the CROSS trial (49% for ESCC) and the 5010 study (43.2%). This may be because our study had more advanced‐stage cancer patients and used different chemoradiotherapy regimens. In the early period, because of concerns regarding the effect of NCRT on surgery, the lymph node drainage area was not included in the radiation field (pCR rate of 19.1%). With the accumulation of clinical experience, the radiotherapy fields generally included lymph node drainage areas, and the pCR rate gradually increased to 36.7%. The pCR rate of 27.9% was similar to that of the recent CMISG1701^25^ study within the normal range.

We found that because of tumor regression, NCIT did not influence the surgical process and may even decrease surgical difficulty. The primary quality control standard for esophagectomy for resecting EC is radical lymph node dissection. A greater number of dissected lymph nodes led to higher OS (hazard ratio [HR] = 0.358; *p* < 0.001) as well as DFS (HR = 0.415; *p* = 0.001); moreover, the lesser number of dissected lymph nodes (<20 vs. ≥20) exhibited a significant association with total recurrence rates (41.2% vs. 25.8%, *p* = 0.027) and increased local recurrence (18.8% vs. 5.2%, *p* = 0.004) in the secondary analysis of the NEOCRTEC 5010 trial.^26^ Our results showed that the NCIT group had more number of harvested lymph nodes than the NCRT group (33.6 vs. 21.7, *p* < 0.001). Following advances in minimally invasive techniques in recent years, an increasing number of patients have undergone minimally invasive esophagectomies. Although there is a lack of concrete evidence regarding survival benefits from techniques such as robot‐assisted surgery, their possible influence on better lymph node dissection cannot be disregarded. The therapy itself may have an influence on the dissection of lymph nodes. In this study, the same lymph node dissection technique was used for most patients. Fibrosis caused by chemoradiotherapy may increase surgical difficulty and lymph node dissection, which may lead to a decrease in the number of lymph nodes removed. A previous study reported similar results wherein NCRT was also associated with fewer dissected lymph nodes (25 vs. 19, *p* < 0.001).^27^

Besides surgical and pathological results, mortality and postoperative complications are critical safety identifiers. It, however, remains unclear whether the incidence of mortality and postoperative complications increases following NCIT in comparison to that after NCRT. Following matching, the overall complication incidence in the NCIT and NCRT groups was 21.2% and 31.8%, respectively. No 30‐day mortality was reported in both groups. Before matching, the NCIT group showed significantly lower anastomotic leakage incidence than the NCRT group (3.8% and 12.5%, respectively; *p* = 0.036). After matching, the incidence of leakage was 4.5% and 10.6%, respectively, with no significant difference; this might be because each group included a smaller number of patients (*p* = 0.188). Local radiation, particularly including cervical esophagus and lymph node fields, is considered a risk factor for postoperative leakage due to decreased vascularity of the tissue and poor healing ability because of tissue edema and fibrosis.

The importance of pCR as the primary endpoint in neoadjuvant therapy remains debatable. A recent international cohort study showed disparities in the prognostic implication of pCR after various neoadjuvant regimens. In the study, the 5‐year RFS rate exhibited a significant decline in patients with pCR after NCRT (75.3%) as compared to those receiving neoadjuvant chemotherapy alone (87.1%, *p* = 0.026). This raises a doubt regarding the credibility of pCR as an oncological marker, particularly for the comparison of neoadjuvant therapy for EC.^28^, ^29^ In our study, the two groups showed comparable pCR rates. After the median follow‐up periods of 27 and 30 months for the NCIT and NCRT groups, respectively, these groups showed 2‐year cumulative OS rates of 80.2% and 62.2%, respectively (*p* = 0.029) and 2‐year DFS rates of 70.0% and 50.8%, respectively (*p* = 0.023). Thus, the present study confirmed that NCIT can provide remarkable short‐term survival benefits as compared to NCRT.

Death and disease recurrence within 1 year after surgery were also analyzed. The NCRT group had more deaths due to tumors than the NCIT group (21.7% vs. 4.6%, *p* = 0.013). The distant and local recurrence rates were 1.5% and 6.2% in the NCIT group and 13.3% and 11.7% in the NCRT group, respectively (*p* = 0.032).

### 4.1 Limitations

Because of the retrospective nature, selection bias and multiplicity must be considered in statistical analysis. PSM was utilized to reduce confounding bias and imbalance between both groups, which increased the reliability of the results. Additionally, the number of included cases was small; moreover, the duration of follow‐up was relatively short, particularly for the NCIT group. Despite this limitation, the follow‐up time covered the high‐risk recurrence period of the first 2 years after surgery. Furthermore, in this study, several immuno‐ and chemo‐regimens were adopted according to the oncologist's preference, and future clinical studies should evaluate these protocols. In recent years, an increasing number of patients have undergone minimally invasive esophagectomies. Although there is a lack of concrete evidence regarding survival benefits from techniques such as robot‐assisted surgery, these techniques might influence the dissection of lymph nodes and overall long‐term patient survival.

## 5 CONCLUSION

As a new treatment option for locally advanced ESCC patients who are undergoing esophagectomy, NCIT achieved superior short‐term survival outcomes as compared to NCRT, with similar perioperative complications and pathological responses. Although NCIT can achieve satisfactory short‐term survival rate, further follow‐up or prospective controlled trials are required to validate whether this short‐term survival ultimately translates into a long‐term survival benefit.^17^

## AUTHOR CONTRIBUTIONS

**Wencheng Zhang:** Data curation (supporting); writing – review and editing (supporting). **Qingsong Pang:** Methodology (supporting); writing – review and editing (supporting). **Abbas E. Abbas:** Methodology (supporting); writing – review and editing (lead). **Hongjing Jiang:** Conceptualization (lead); investigation (lead); project administration (lead); writing – original draft (equal); writing – review and editing (lead). **Xiaobin Shang:** Conceptualization (supporting); data curation (supporting); writing – review and editing (supporting). **Fangdong Zhao:** Data curation (equal); formal analysis (equal); writing – original draft (equal). **Chen Zhang:** Data curation (supporting); writing – review and editing (supporting). **Jie Yue:** Formal analysis (supporting); writing – review and editing (supporting). **Zuoyu Chen:** Formal analysis (supporting); writing – review and editing (supporting). **Zhao Ma:** Data curation (supporting); writing – review and editing (supporting). **Chuangui Chen:** Data curation (supporting); writing – review and editing (supporting). **Xiaofeng Duan:** Conceptualization (equal); data curation (equal); formal analysis (equal); methodology (equal); writing – original draft (equal); writing – review and editing (equal).

## CONFLICT OF INTEREST STATEMENT

All authors have nothing to disclose.

## Data Availability

The data that support the findings of this study are available on request from the corresponding author. The data are not publicly available due to privacy or ethical restrictions.
